# Age and environment-related differences in gait in healthy adults using wearables

**DOI:** 10.1038/s41746-020-00334-y

**Published:** 2020-09-30

**Authors:** Matthew D. Czech, Dimitrios Psaltos, Hao Zhang, Tomasz Adamusiak, Monica Calicchio, Amey Kelekar, Andrew Messere, Koene R. A. Van Dijk, Vesper Ramos, Charmaine Demanuele, Xuemei Cai, Mar Santamaria, Shyamal Patel, F. Isik Karahanoglu

**Affiliations:** Early Clinical Development, Pfizer, Inc., Cambridge, 02139 MA USA

**Keywords:** Predictive markers, Quality of life, Biomedical engineering

## Abstract

Technological advances in multimodal wearable and connected devices have enabled the measurement of human movement and physiology in naturalistic settings. The ability to collect continuous activity monitoring data with digital devices in real-world environments has opened unprecedented opportunity to establish clinical digital phenotypes across diseases. Many traditional assessments of physical function utilized in clinical trials are limited because they are episodic, therefore, cannot capture the day-to-day temporal fluctuations and longitudinal changes in activity that individuals experience. In order to understand the sensitivity of gait speed as a potential endpoint for clinical trials, we investigated the use of digital devices during traditional clinical assessments and in real-world environments in a group of healthy younger (*n* = 33, 18–40 years) and older (*n* = 32, 65–85 years) adults. We observed good agreement between gait speed estimated using a lumbar-mounted accelerometer and gold standard system during the performance of traditional gait assessment task in-lab, and saw discrepancies between in-lab and at-home gait speed. We found that gait speed estimated in-lab, with or without digital devices, failed to differentiate between the age groups, whereas gait speed derived during at-home monitoring was able to distinguish the age groups. Furthermore, we found that only three days of at-home monitoring was sufficient to reliably estimate gait speed in our population, and still capture age-related group differences. Our results suggest that gait speed derived from activities during daily life using data from wearable devices may have the potential to transform clinical trials by non-invasively and unobtrusively providing a more objective and naturalistic measure of functional ability.

## Introduction

Gait is the primary means of mobility for most individuals and many conditions directly or indirectly have an impact on gait. Gait speed is considered an informative and reliable clinical measure in a wide range of disease populations; it is often referred to as the sixth vital sign^1,2^. Previous studies have shown that lower gait speed is associated with cognitive decline, falls, and mortality^3,4^, and is an important indicator of health and function in ageing and disease^5^. Conventional gait assessment is performed in the laboratory or clinic, using a combination of observational scales (e.g., functional gait assessment) and performance tests (e.g., 6 min walk test), where individuals perform prescribed walking tests under observation. These types of assessments may not be able to provide a reliable estimate of real-world gait because (1) they are administered episodically, (2) can be subjective in nature, and (3) gait can be altered under observation (Hawthorne effect)^6,7^.

Advances in wearable technology have enabled the measurement of gait using inertial sensors in free-living conditions^8^. Using ground truth references, such as instrumented mats and motion capture systems, researchers have validated novel gait measurement approaches that rely on a small number of battery-efficient inertial sensors^9,10^. Comparative analysis has revealed that while these methods generally perform well, there are considerations, such as study population and device location that can influence the reliability of measurements^11,12^. Therefore, the validity of such methods needs to be rigorously assessed in different populations to establish their performance characteristics. In addition to assessing the accuracy with which they can measure spatial and temporal aspects of gait, it is also necessary to evaluate the sensitivity of measures derived during daily life for detecting clinically meaningful changes. Shah et al. showed that measures of quantity were able to better discriminate between patients with multiple sclerosis and controls, whereas measures of quality were more discriminative for patients with Parkinson’s disease (PD) and controls^13^.

There is a growing body of research showing that gait assessments performed under controlled conditions (e.g., in the laboratory or clinic) are unable to capture the variability observed during daily life (e.g., in the home and community)^14–17^. Specifically, gait speed derived from data captured under continuous free-living conditions is slower than gait speed measured in the clinic in frail elderly or community-dwelling older adults^14^. It has been hypothesized that continuous, at-home monitoring provides a richer and more comprehensive view of an individuals experience with the disease^18,19^. In fact, Del Din et al. showed that distinguishing PD patients and healthy volunteers using gait characteristics was improved in free-living conditions^20^. In addition to the prospect of enhanced sensitivity of measurements, at-home monitoring has the potential to improve patient engagement in clinical research studies by reducing the need for frequent visits to the clinic, enabling more patients to participate, and reducing the burden on patients and caregivers. For these reasons, at-home measurements are gaining traction as valid clinical endpoints from regulatory agencies. For example, the European Medicines Agency recently approved 95^th^ percentile stride velocity measured using a valid and suitable wearable device, as an acceptable secondary endpoint in pivotal or exploratory clinical studies for Duchenne muscular dystrophy^21^.

Despite growing evidence that at-home monitoring provides a more comprehensive assessment of gait, several hurdles need to be addressed to enable broader clinical adoption. Further clinical research that adopts and validates standardized sensor-based methods in various populations under free-living conditions is needed to translate research findings and novel methods into practice^22^. In addition, questions remain regarding the processing and interpretation of at-home data^23^. An open question is the optimal monitoring duration necessary for reliable characterization of gait under free-living conditions. Obtaining data from multiple days and investigating day-to-day variability of at-home measures is necessary, in order to assess the minimum required acquisition period and obtain reliable real-world estimates. However, additional days of monitoring results in increased patient burden and might reduce compliance in clinical trials, especially for patients suffering from particularly debilitating diseases. The required number of at-home monitoring days still remains arbitrary and can be affected by multiple factors, such as the type of disease, treatment, age, geographical location, and socioeconomic status. In fact, previous studies have used data captured during monitoring durations that ranged from one day to several weeks for their analysis^20,24–27^. Several studies have also investigated day-to-day variability of at-home measures^27–33^. These studies have reported a minimum of three to six days of measurements required to obtain reliable estimates of physical activity, energy expenditure, and heart rate. However, the monitoring duration necessary for deriving a reliable estimate of gait speed under free-living conditions is not well understood. We are aware of only one recent study that reported a minimum monitoring duration of 3 days for reliable estimation of gait speed in slow-walking older adults with sarcopenia^17^.

Herein, we present our work on assessment of gait in healthy younger (18–40 years) and older (65–85 years) adults in both the laboratory and home setting, using a single lumbar-worn wearable accelerometer. We aim to (1) assess the validity of measurements derived using the lumbar-worn wearable device by comparing them with those provided by a system that use multiple wearable devices (APDM) and an instrumented mat (GAITRite), (2) test the sensitivity of the median and 95^th^ percentile gait speed derived from in-lab walk test and continuous at-home monitoring data to detect age-related group differences, and (3) propose a minimal at-home monitoring period for estimating gait speed reliably.

## Results

### Gait speed can be derived accurately from single lumbar-worn accelerometer

**Fig. 1:**
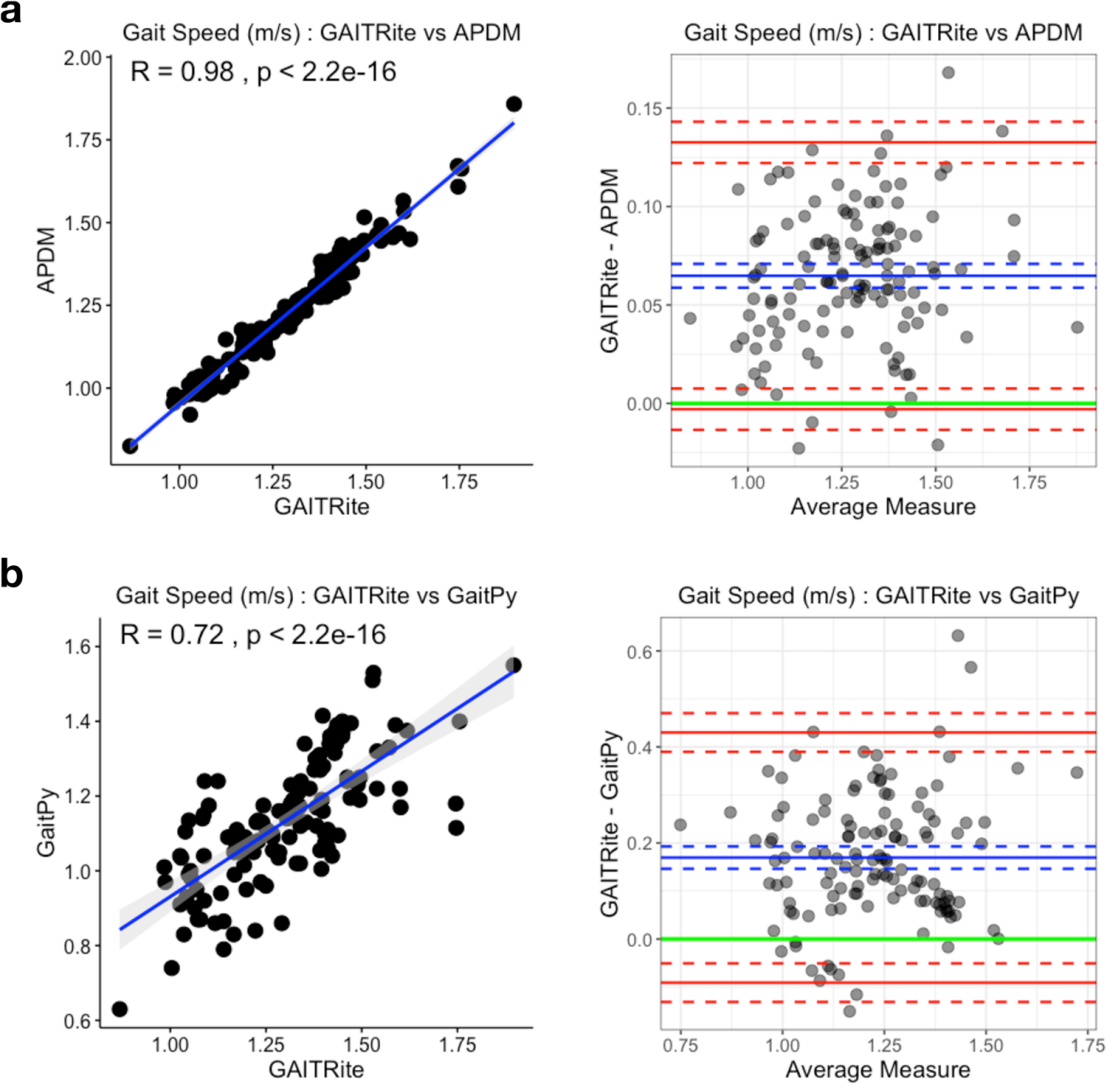
Gait speed validation based on in-lab 4-m gait task. **a** Comparison of gait speed estimated using a six-sensor system (APDM) and an instrumented gait mat (GAITRite). The gait speeds derived from two systems were highly correlated (Pearson’s *r* = 0.98, left). Bland–Altman plots (right) showed minimal mean difference (mean difference = 0.07, blue solid line; LoA = [−003, 0.13], red solid lines; corresponding confidence intervals are in dashed lines). **b** Comparison of gait speed estimated using a single lumbar-worn sensor (GaitPy) and an instrumented gait mat (GAITRite). The gait speeds derived from two systems were also highly correlated (Pearson’s *r* = 0.72, left). Bland–Altman plots (right) showed mean difference (mean difference = 0.17, blue solid line; LoA = [−0.09, 0.43] red solid lines; corresponding confidence intervals are represented by dashed lines). Both APDM and GaitPy had consistent bias compared to GAITRite and underestimated gait speed. LoA limits of agreement.

During in-lab assessments, participants walked three 4-m laps on an instrumented mat (GAITRite) at their typical walking speed. While performing the task, participants wore six devices (Opal and APDM), which were located at the sternum, lumbar, and bilaterally on the wrists and feet. We assessed the accuracy and reliability of gait features derived using (1) the APDM (six-sensor set) method^34^ and (2) the GaitPy (single lumbar-mounted sensor) method^35^, by comparing them with gait features provided by GAITRite (considered here as the gold standard). Figure 1 depicts the agreement of gait speed derived from APDM and GaitPy, with respect to the GAITRite through correlation and the Bland–Altman plots. The intraclass correlation coefficient (ICC) between gait speed derived using the three methods showed moderate agreement (ICC = 0.66, lower and upper bounds = [0.27–0.83]). While GaitPy had higher variability than APDM measurements (GAITRite vs GaitPy ICC = 0.49, lower and upper bounds = [−0.07–0.77], after mean bias correction ICC = 0.72, lower and upper bounds = [0.63–0.79]), both APDM and GaitPy had good agreement with GAITRite. The distributions of both APDM- and GaitPy-derived gait speeds were homoscedastic with consistent mean biases with respect to GAITRite (GAITRite − APDM = 0.07 m/s (5%), GAITRite − GaitPy = 0.17 m/s (13%)).

### In-lab gait speed did not distinguish between age groups

**Fig. 2:**
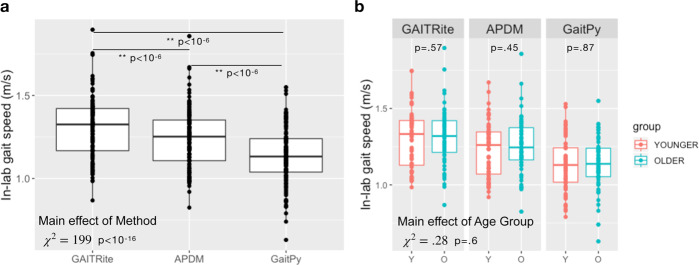
In-lab gait speed did not show any age group differences. **a** Gait speed estimated using different methods differed (*χ*^2^ = 199, *p* < 10^−16^). Both APDM and GaitPy underestimated gait speed during in-lab gait task compared to GAITRite (*p* < 10^−6^), which is used as the gold standard. **b** Gait speed estimated using any of the three methods did not differ between the two age groups (younger group, *n* = 33, age = 29.2 ± 4.6, 17F; older group, *n* = 32, age = 72.3 ± 5.8, 16F; main age group effect: *χ*^2^ = 0.28, *p* = 0.6). Box and whiskers plots show the median and interquartile range, the lines extend to the smallest/largest value within 1.5 times interquartile range below/above the 25th/75th percentile, and the dots represent each individual data value.

In order to test if gait speed differs between the younger and older age groups (younger group, *n* = 33, age = 29.2 ± 4.6 years; older group, *n* = 32, age = 72.3 ± 5.8 years, full demographics in Table 1), we first performed group analysis on the in-lab gait speed. The overall repeated measures regression model included methods (GAITRite/APDM/GaitPy), age group (younger/older), visit (visit1/visit2), sex (F/M), height, and muscle mass as independent variables (fixed effects) and subject (random effect). The average gait speed estimated by different methods was significantly different (main effect of method: *χ*^2^ = 199, *p* < 10^−16^; Fig. 2a). Pairwise comparisons further showed that both APDM (six-sensor) and GaitPy (single lumbar sensor) underestimated in-lab gait speed compared to GAITRite (*p* values of all pairwise comparisons <10^−6^). There was no main effect of age group (*χ*^2^ = 0.28, *p* = 0.6). When pairwise comparisons of the age group differences were tested, none of the methods were able to differentiate between younger and older groups (Fig. 2b). There were a trending main effect of visit (*χ*^2^ = 3.79, *p* = 0.051), significant age group by sex interaction (*χ*^2^ = 5.43, p = 0.02), and age group by sex and by method interaction (*χ*^2^ = 14.67, *p* < 10^−3^). No other variables or covariates had significant effects on gait speed.

**Table 1:** Participant demographics.

	Younger	Older	*p* Value
Number of participants	33	32	
Sex (F/M)	17/18	16/18	1
Age (years)	29.2 ± 4.6	72.3 ± 8.8	
	[23–39]	[65–85]	
BMI (kg/m^2^)	23.4 ± 2.6	24.5 ± 2.6	0.9
	[19–29]	[19–29]	
Education	13 College	3 High school	0.14
	20 Postgrad	7 College	
		22 Postgrad	
Ethnicity	25 White	30 White	0.07
	6 Asian	1 Asian	
	2 Other	1 Other	

In total, 65 participants were included in the study, which was conducted at the Pfizer Innovation Research Laboratory (PfIRe Lab), MA. The younger and older age groups were equibalanced in terms of sex and BMI. The younger group included one American Indian or Alaska Native, and one Native Hawaiian or Pacific Islander; the older group included one Black or African American participant, which were included in the “other” category.

*BMI* body mass index.

### At-home gait speed differed significantly between age groups

**Fig. 3:**
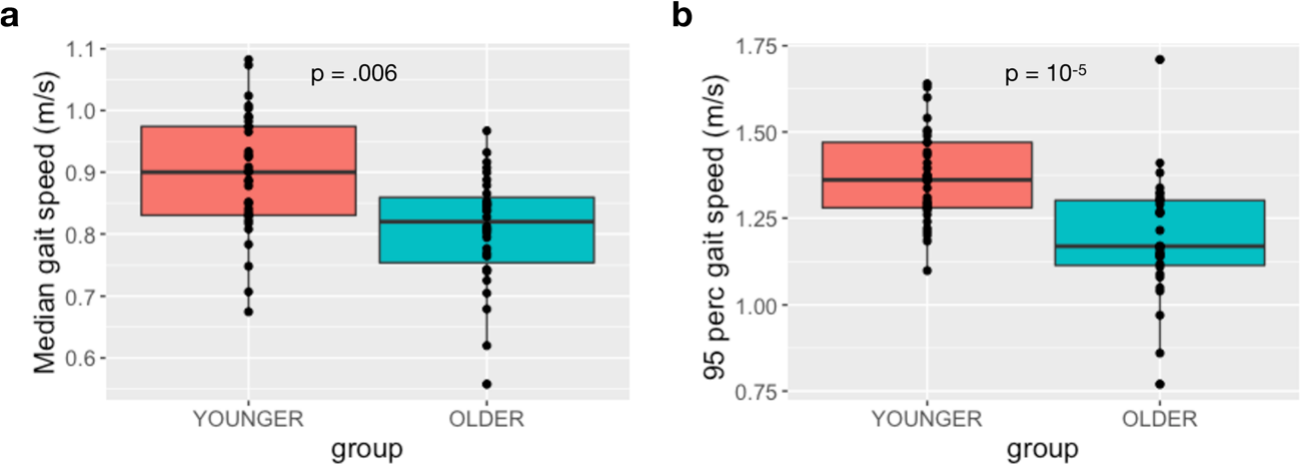
At-home gait speed estimated using a single lumbar-worn sensor (GaitPy) differed between age groups. **a** The median gait speed estimated by GaitPy showed significant group differences between younger and older groups (*p* = 0.006). There was also significant main effect of day type (*χ*^2^ = 42.08, *p* < 10^−5^), and group by day type interaction (*χ*^2^ = 13.38, *p* = 0.002); i.e., the group difference was larger during weekdays than weekends. **b** The 95^th^ percentile gait speed was also different between younger and older groups (*p* = 10^−5^). Box and whiskers plots show the median and interquartile range, the lines extend to the smallest/largest value within 1.5 times interquartile range below/above the 25th/75th percentile, and the dots represent each individual data value.

Participants were asked to continuously wear an accelerometer (GeneActiv) attached to the lumbar region with an elastic belt for a period of approximately one week (range = [6–15] days, mean ± SD = 8.72 ± 1.88 days; younger group = 8.61 ± 1.73 days; older group = 8.84 ± 2.05 days. There were no group differences between the number of walking bouts of younger and older groups during the at-home monitoring period (*p* = 0.8). We then performed group analysis on at-home gait speed of the two age groups. The linear mixed-effects model showed significant age group differences for both median gait speed (*χ*^2^ = 12.54, *p* = 0.006) and the 95^th^ percentile gait speed (*F* = 22.59, *p* = 10^−5^) between the younger and older groups Fig. 3a, b, respectively). The older group walked significantly slower than the younger group. There was a significant effect of day type (weekday/weekend, *χ*^2^ = 42.08, *p* < 10^−5^) as well as a group by day type interaction for median gait speed (*χ*^2^ = 13.38, *p* = 0.002), indicating that the age group difference was larger during weekdays than weekends. The pairwise comparisons showed significant age group differences for weekdays (*χ*^2^ = 21.81, *p* < 10^−4^), but not for weekends (*χ*^2^ = 3.23, *p* = 0.33). There were no other effects of covariates or interactions.

### Weak association between at-home and in-lab gait speed

**Fig. 4:**
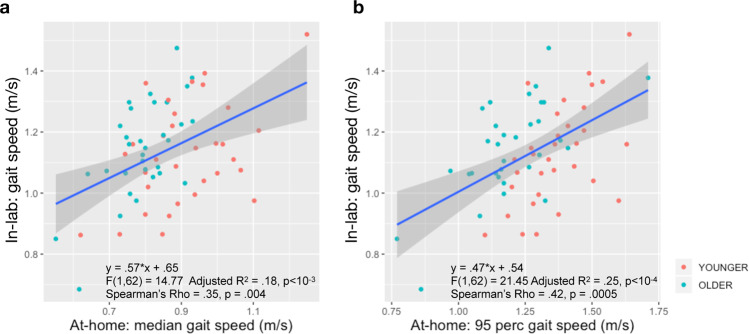
Weak association between in-lab and at-home gait speed. **a** The median gait speed at home showed a significant slope and an intercept (*β* = 0.57, *p* < 10^−3^, *I* = 0.65; *p* < 10^−5^). The two gait speed measures were moderately correlated (Spearman’s rho = 0.35, *p* = 0.004), and at-home median gait speed explained only 18% of the variance of in-lab gait speed. **b** When a regression analysis was performed to explain the in-lab gait speed with the 95^th^ percentile gait speed, at-home gait speed showed a significant slope and an intercept (*β* = 0.47, *p* < 10^−4^; *I* = 0.54, *p* = 10^−4^). The two gait speed measures showed moderate correlation (Spearman’s rho = 0.42, *p* = 0.0005). At-home 95^th^ percentile gait speed explained only 25% of the variance of in-lab gait speed. Shaded area shows the 95% confidence interval.

We evaluated the agreement between in-lab and at-home gait speed, both estimated from the lumbar sensor using the GaitPy method^35^. Separate regression analyses were used to test if the median and the 95^th^ percentile gait speed at home predicted the in-lab gait speed. Although both the median and 95^th^ percentile gait speed at home significantly predicted the in-lab gait speed, they only explained ~20% of the variance with significant intercept (median: adjusted *R*^2^ = 0.18, *F*(1,62) = 14.77, *β* = 0.57, *p* < 10^−3^, 95^th^ percentile: adjusted *R*^2^ = 0.25, *F*(1,62) = 21.45, *β* = 0.47, *p* < 10^−5^). Correlation analysis showed only a moderate relationship between at-home and in-clinic gait speed metrics (Spearman’s rho = 0.35 and 0.42, Fig. 4).

### Data from three days at home are sufficient for estimating gait speed

**Fig. 5:**
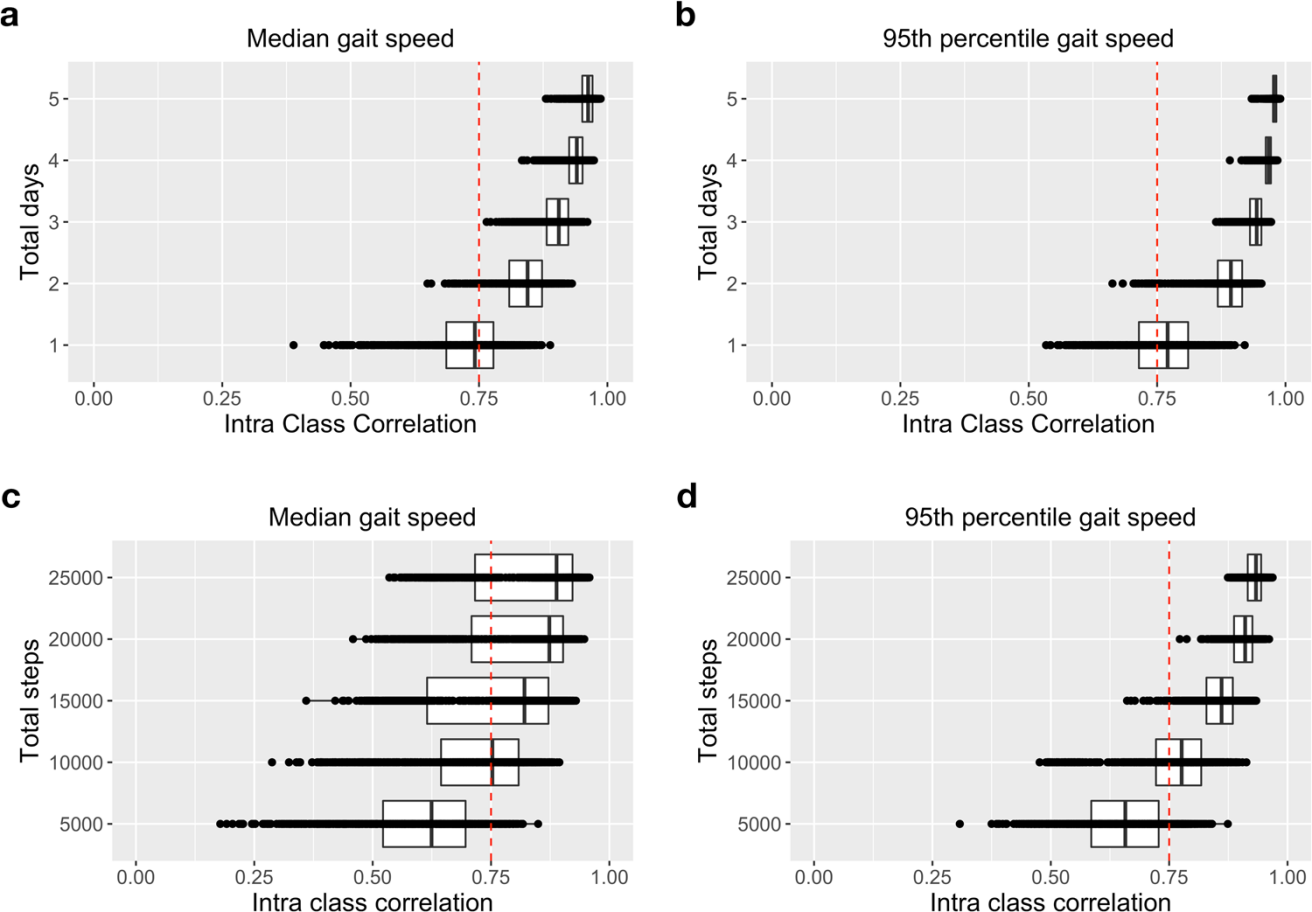
Amount of data needed to reliably estimate gait speed at home. Subset of data in terms of successive steps or randomly selected days was compared to the full data set (ICC > 0.75 represents excellent agreement between two measurements). Minimum required data to estimate both **a** median gait speed and **b** 95^th^ percentile gait speed was 2–3 days of monitoring data. **c**, **d** At least 15,000 and 10,000 concurrent steps were required to reliably estimate median and 95^th^ percentile gait speed, respectively. Box and whiskers plots show the median and interquartile range, the lines extend to the smallest/largest value within 1.5 times interquartile range below/above the 25th/75th percentile, and the dots represent each individual data value.

We investigated the minimum amount of data (in terms of steps or days) required from at-home monitoring, in order to reliably estimate gait speed. Median ICC was used to assess agreement between gait speed estimated using subsets of data (bootstrap with replacement across various days of monitoring) and gait speed estimated using full data set (i.e., all available days). Compared to the full data, the agreement between two days or more, and full data were excellent (ICC > 0.75) for both median (ICC = 0.85, [0.65–0.93]) and 95^th^ percentile gait speed (ICC = 0.89, [0.66–0.95], Fig. 5a, b). The improvement on ICC values becomes minimal starting from three days with ICC > 0.75 for all bootstraps. Moreover, good agreement was observed for both median and 95^th^ percentile gait speed for 5000 steps, and excellent agreement was observed for both median and 95^th^ percentile gait speed, when participants walked 15,000 or more successive steps, though there was substantial variance, especially for median gait speed (Fig. 5c, d). The improvement in 95^th^ percentile gait speed becomes minimal starting from 20,000 steps.

Based on these findings, we investigated the impact of monitoring duration on detecting differences between the two age groups based on at-home measures of gait speed. We applied a *t*-statistic bootstrapping method^36^ to create various subsets of days, and compared them to the entire data set collected outside the lab. Similar to the results above, differences between median gait speed for the two age groups were significant with data from only two at-home days (all at-home data vs two days: *p* ≥ 0.05, Table 2). Moreover, for 95^th^ percentile gait speed, distinguishability between younger and older groups for 1 day of data were comparable to full data set obtained outside the lab (all at-home data vs 1 day: *p* > 0.05, Table 2).

**Table 2:** The minimum number of at-home monitoring days required to differentiate the at-home gait speed between younger and older groups similar to full data.

Days	Median gait speed			95^th^ Percentile gait speed		
	*t*-Original	*t*-Bootstrap^b^	*p* Value	*t*-Original	*t*-Bootstrap^b^	*p* Value
1		3.01 ± 0.67	0.048		3.35 ± 0.68	0.08^a^
2		3.57 ± 0.51	0.146^a^		3.73 ± 0.53	0.147^a^
3		3.79 ± 0.42	0.225^a^		3.96 ± 0.41	0.214^a^
4		3.91 ± 0.33	0.259^a^		4.06 ± 0.33	0.243^a^
5		3.99 ± 0.26	0.292^a^		4.15 ± 0.26	0.288^a^
all	4.13			4.29		

For both 95^th^ and median gait speed 1000 bootstraps were drawn for various number of days [1–5] and the *t*-values were compared to the original *t*-values. In order to obtain similar group distinguishability compared to full data set, at least 2 and 1 days of at-home monitoring data with 1000 steps were required for median and 95^th^ gait speed, respectively (*p* > 0.05). *t*-Original shows the *t*-statistic for the group difference using the full data, *t*-bootstrap indicates the mean *t*-statistic over the bootstrap samples.

^a^*p* < 0.05.

^b^Mean ± sd.

## Discussion

In this cross-sectional study involving healthy adults divided into two age groups (younger [18–40 years], older [65–85 years]), we derived gait speed from participants during in-lab gait tasks, as well as from approximately nine days of continuous at-home monitoring. We aimed to (1) validate and evaluate the performance of a method for measuring gait relying on a single lumbar-worn accelerometer (GaitPy) with respect to a reference method relying on six devices (APDM), and an instrumented mat (GAITRite) as the gold standard device in-lab; (2) test the ability of gait speed estimated in lab and at home to distinguish between the two age groups; (3) determine the amount of at-home data required to reliably estimate the median and 95^th^ percentile gait speed, and (4) evaluate the sensitivity of gait speed measured at home for detecting age-related differences in a healthy population.

Assessing the validity of sensor-based methods for estimating gait speed in a controlled setting (e.g., laboratory or clinic) is essential for understanding its performance characteristics. The algorithm we implemented in GaitPy for estimating gait speed using a single lumbar-worn sensor has been shown to be comparable to gait measures derived using bilaterally worn ankle-mounted devices, and has previously been successfully applied in a variety of disease populations, including PD, Huntington’s disease, and stroke patients, both in-lab and at-home^11,12,37,38^. Compared to shin or foot sensors, a single lumbar-worn sensor enables relatively easy removal and reapplication during certain periods of the day, including bathing and sleeping. In addition, the lumbar position is a convenient location for measuring bilateral asymmetries of gait which may be important in certain disease populations, including PD. In this study, we confirmed the validity of a lumbar-worn sensor and observed that in-lab gait speed estimated using data from a single lumbar-worn sensor (GaitPy) showed good agreement with an instrumented mat (GAITRite). We further observed a consistent bias for both GaitPy (13%) and APDM (5%, reference six-device system) compared to the instrumented mat. However, GaitPy-derived gait speed had higher variability than APDM, likely the result of reliance on a single device. This suggests that there might be a sensitivity trade-off when utilizing fewer devices for measuring gait speed. Although, based on our previous findings, this trade-off may be minimal^39^. Using gyroscope in combination with the accelerometer could potentially lead to better gait characterization, especially important to capture rotational information for turns and falls, with a cost of higher battery usage. Despite limitations in sensitivity, gait speed estimated using a single lumbar-worn device has been shown to distinguish between disease states, as well as detect the effects of treatment^20^. Additional work investigating the validity of gait feature estimation using a single lumbar-mounted device in various disease populations is still needed. Our results suggest that a single lumbar-worn device can provide sufficient accuracy for monitoring gait under free-living conditions and at the same time minimize participants burden.

There is mounting evidence that gait measurements differ between in-lab and at-home environments^14–17^. In our study, in-lab gait speed derived using either wearable sensors or GAITRite was unable to distinguish between the two age groups, whereas at-home gait speed showed the older group walked significantly slower than younger participants. We further confirmed our findings using uninstrumented measurements, which were collected as part of the traditional Short Physical Performance Battery (SPPB) assessment on the same participants (i.e., the time for participants to perform the walk test timed using a stop watch), and found no differences between age groups based on stop watch time (average time to walk 4-m, younger group: 3.8 ± 0.5 s, older group: 3.7 ± 0.5 s, *χ*^2^ = 1.08, *p* = 0.3). Furthermore, other gait metrics such as step time and stride length derived from in-lab assessments failed to differentiate the age groups (Supplementary Table 1). In-lab measures are acquired during single visits, whereas at-home measurements enable continuous evaluation over prolonged periods, providing the ability to capture nuanced therapeutic effects^18^. Our findings are consistent with evidence that while a participant might change his/her behavior for a short period of time under observation (e.g., no age group difference for gait speed during in-lab assessments), it is unlikely that they will be able to do so during long periods of passive monitoring under free-living conditions^14^.

We have not observed a sex effect, but there was an age group by sex interaction during the short in-lab walk test captured by both instrumented and uninstrumented measurements (Supplementary Fig. 1). This effect did not exist in the at-home monitoring data, and we suggest that the interaction effect is due to the participants change of behavior during short, observed assessments in the lab environment (i.e., observer effect). Therefore, healthy volunteer studies interested in at-home gait speed as an endpoint may not need independent grouping based on sex. However, controlling for age may be needed in studies with wide age ranges.

Several studies have proposed that gait should be considered as the sixth vital sign^2^. For example, higher gait speed predicted better survival^40^ and lower gait speed was associated with serious falls that resulted in a visit to the emergency room in older adults, with mild cognitive impairment^41^. In healthy adults, real-world gait speed is expected to decrease approximately 0.03 m/s every decade over the life span of an adult, resulting in a difference of around 0.12 m/s after 40 years^42^. In our study, we observed that at-home gait speed differed by 0.09 m/s on average between the younger and older groups (approximately four decades apart), and that group difference in median gait speed was driven by weekday rather than weekend periods (Supplementary Fig. 2a). We also observed that age-based differences existed for various bout length, especially for short (ranging between 10 and 30 s) and medium bouts (ranging between 30 and 60 s), although gait speed increased with bout length (effect of age group: *χ*^2^ = 6.62, *p* = 0.02, effect of bout length: *χ*^2^ = 557, *p* < 10^−16^ Supplementary Fig. 2b). Furthermore, not only gait speed but other gait features differed by age group (Supplementary Table 2). Similar trend was observed in patient populations such as PD, where significant group differences in at-home gait features were observed compared to healthy volunteers^20^. Although these are cross-sectional studies, accurate estimation of real-world gait and evaluating its sensitivity to clinically meaningful change; e.g., the disease state or its progression, are extremely important. We have shown that gait metrics derived from at-home monitoring with a single lumbar-worn sensor provided more sensitive information to differentiate two age groups compared to in-clinic assessments, and that there is only a weak association between at-home and in-lab gait speed. This result was recently reported in older adults^14,15,17^, and it was also shown that gait speed derived during longer walking bouts in the laboratory appears to be better correlated with at-home measurements^17^. These findings provide further evidence for implementing wearable devices in clinical trials for monitoring physical function at home instead of in-clinic assessments. The use of wearable devices out-of-lab settings not only provides a better assessment of real-world activity, but also brings additional benefits of remote monitoring. For example, it enables to enroll patients who live in remote regions with difficult access to clinic, or reduces the exposure of participants who may be vulnerable to healthcare settings during a pandemic. In summary, remote data capturing offers the ability to design decentralized trials, which may become crucial for a wide variety of clinical trials in the near future.

Overall, the median gait speed measured at home was lower than in-lab gait speed for both age groups. This result suggests that at-home gait speed may be affected by one or more factors that are present during daily life. One possible factor may be the cognitive challenges that are typically present during performance of activities of daily living. Several studies suggest that mobility relies on cognitive resources^43–45^. In fact, it has been recently shown that mobility assessments performed at home better reflect cognitive functioning compared to those performed in the laboratory^46^. In addition, Hillel et al. showed that gait speed measured during in-lab dual-task walking is comparable to the gait speed measured during at-home monitoring^15^. The presence of a relationship between cognition and mobility performance may explain the reduced at-home gait speed compared to in-lab gait speed we observe in our data. Additional factors such as mood and fatigue may also contribute to the at-home gait, more so than in lab, where participants may put forth their best effort.

Our results suggest that at least two to three days of monitoring is required to estimate both median and 95^th^ percentile gait speed in healthy volunteers. When the age groups were analyzed separately, we found only one to two days of data was needed to reliably estimate gait speed in the older cohort, whereas two to three days were needed for the younger cohort. This result suggests more day-to-day variance in gait speed in the younger cohort (Supplementary Fig. 3), as also reflected by the significant effect of day type, as well as age group by day type interaction (Supplementary Fig. 2a). In addition, our results (Table 2) suggest that two days of data were necessary to estimate differences between young and old using median gait speed, whereas one day was needed for 95^th^ percentile gait speed. Indeed, the upper and lower ICC bounds were tighter for 95^th^ percentile gait speed compared to median gait speed, suggesting less day-to-day variability of 95^th^ percentile gait speed. The lower variability of 95^th^ percentile gait speed could have contributed to the improved distinguishability of young and older groups with less data compared to median gait speed. It may also be the case that median gait speed is a more sensitive indicator of age. A previous study employed a similar approach to quantify the minimum days required for reliable estimation of physical activity in older adults, and found that two days were sufficient for seven of the nine activities^32^. In accordance with our results, another study in older adults found that three days of accelerometer data were needed to accurately predict physical activity levels^28^, and another recent study found that a monitoring period of three days is necessary for gait speed estimation in frail older adults^17^.

Our results on healthy participants comparing two age groups will be helpful for future studies with multiple disease populations; e.g., for selecting important variables, deciding the test environment, and minimal monitoring period. Moreover, we suggest this study provides an evidence on the ability of gait speed to detect minimal change between two close populations, especially important during the early stages of gait impairment, where only subtle differences may be detected relative to healthy participants, and during disease progression. However, validation of GaitPy performance in advanced disease populations may be needed to verify accuracy of the algorithm. In addition, disease-specific effects, such as motor fluctuations seen in some PD patients due to wearing off medication, could produce changes in variability of at-home gait speed. Therefore, future work would be needed to consider the potential impact of disease-specific changes on algorithm reliability and gait speed variability.

One limitation of our study is that we measured gait speed in healthy adults for just over 1 week, (mean ± SD = 8.72 ± 1.88 days). The group analyses were conducted based on the full data; however, we limited gait speed reliability analysis to participants with at least five days of data (number of participants who had five days of data with at least 1000 steps per day = 64, number of participants who had five days of data with at least 100 steps per day = 65). Although we instructed our participants to wear the device continuously, we did not have a robust way to determine participant compliance for a lumbar-worn accelerometer. Therefore, we set a minimum threshold of 100 steps for including a day in our analyses. We investigated different step threshold values (10, 250, and 1000 per day), but they did not have any significant impact on the results (Supplementary Fig. 4). Accurate determination of participant compliance remains a challenge in the field and a limitation of this study.

Another limitation of this analysis is that the randomly selected days, varying from 1 to 5 days, were compared to the full data set, which captured on average 9 days of at-home monitoring. Moreover, we have observed that the type of day (weekend/weekdays) has a significant effect on gait metrics (Supplementary Table 2 and Supplementary Fig. 2a). In our analysis, we only tested for steps or days without labeling the type of day, since that would introduce another limit for bootstrapping. Further analysis accounting for the day type showed that including weekend day out of a total of 3 days only slightly improved reliability of both median and 95^th^ percentile gait speed estimation (Supplementary Fig. 5). Future studies may benefit from longer monitoring periods, in order to obtain more substantial baseline data to replicate these findings.

In the present study, we have shown that a single lumbar-worn sensor can be used for monitoring gait under free-living conditions and capture meaningful information about real-world function that might not be possible in controlled settings (e.g., laboratory or clinic). We have shown that, despite higher variability, at-home gait speed was able to capture age-related group differences in healthy volunteers. In contrast, in-lab gait speed measured using either of the three methods did not differentiate between the two age groups. Moreover, we found that there was a weak correlation between at-home and in-lab gait speed, and gait speed measured at home was lower than in lab for both age groups. Finally, two to three days of at-home monitoring is sufficient for reliably estimating median and 95^th^ percentile gait speed in both older and young healthy adults.

## Methods

### Subjects and procedure

We recruited 65 participants in total, 33 healthy young participants (age = 29.2 ± 4.6 years, 17F, body mass index (BMI) = 23.4 ± 2.6) and 32 healthy older participants (age = 72.3 ± 5.8 years, 16F, BMI = 24.5 ± 2.6, Table 1) to take part in two instrumented in-lab assessments each lasting around two hours in duration about 7–14 days apart, and an at-home portion in between the two visits (range = [6–15] days, mean ± SD = 8.72 ± 1.88 days; younger group = 8.61 ± 1.73 days; older group = 8.84 ± 2.05 days). Throughout this manuscript at-home activity monitoring refers to monitoring all activity outside the laboratory; i.e., real-world environment or daily life.

The in-lab portion was completed at the Pfizer Innovation Research Lab (PfIRe Lab) in Cambridge, Massachusetts. The study was reviewed and approved by Advarra IRB (protocol number: Pro00029419). All participants signed the written informed consent. The eligibility criteria included no significant health problems, as reviewed by the study physician during medical history intake; BMI ≥ 18.5 (kg/m^2^) and <30 (kg/m^2^) or absolute weight <125 kg; and the predetermined score for VES-13 (Vulnerable Elders Survey).

During the in-lab portion, participants were instrumented with six wearable inertial devices (Opal, APDM Inc., Portland, Oregon) consisting of three-axis accelerometer, gyroscope, and magnetometer worn on the sternum, lumbar (L4 position), and bilaterally on the wrists and feet. The devices recorded data from three-axis accelerometer, gyroscope, and magnetometer at a sampling rate of 128 Hz. Subjects were asked to complete a battery of activities, including sit-to-stand tasks, postural/balance tasks, and a gait task, as part of the SPPB assessment. The analysis presented herein was limited to data from the gait task during which participants walked three laps on an instrumented mat (GAITRite, CIR Systems Inc., Franklin, New Jersey), while wearing the APDM six-sensor set. Uninstrumented measurements were also acquired using stop watch to register the time to complete the tasks as part of the standard SPPB assessment.

For the at-home portion, participants were instructed to wear a device (GENEActiv, Activinsights Ltd., UK) on their lower back and wrist continuously for a period of about 7–14 days. Both devices recorded three-axis accelerometer data at sampling rate of 50 Hz. Only accelerometer data from the lumbar-worn device was used for the analysis presented herein.

### Gait feature extraction

Three separate methods were used for estimating gait speed during the performance of a gait task in the laboratory. Ground truth gait speed was estimated from data collected using an instrumented mat (GAITRite), using a vendor supplied proprietary algorithm (GAITRite Software version 4.8.5). In addition, six inertial sensors (Opal, APDM) located on the sternum, lower back, and bilaterally on the wrists and feet, were used to estimate gait speed using a vendor supplied proprietary algorithm (APDM Mobility Lab v2.0.0.2018). Lastly, we estimated gait speed from accelerometer data recorded using a lumbar-mounted device (Opal, APDM), using an open-source algorithm (GaitPy v1.6.0), we implemented in Python v3.6^35^. GaitPy uses a wavelet-based method to enhance patterns that occur in the vertical acceleration signal for first detecting heel strike and toe off events during a gait cycle^43^. Gait speed is then estimated by integrating the vertical acceleration signal to derive vertical displacement and applying an inverted pendulum model as described by Zijlstra et al.^47^.

GaitPy was also used to estimate gait speed from data collected at home. For at-home data, GaitPy first uses a binary classifier to detect bouts of gait. Bouts of gait <3 s apart are concatenated into a single bout before estimating gait speed on a stride by stride basis.

### Statistical analysis

Statistical analysis was performed in R version 3.5.2 with following main packages: “lme4” for linear mixed-effect regression, “car” for type-III ANOVA, “BlandAltmanLeh” for Bland–Altman plots, and “psych” for ICC. For in-lab walk test, for each digital device and algorithm aforementioned, the median of gait metrics across all steps for each lap was computed. Then, the median values across all laps per visit were used for statistical analysis. Bland–Altman plots were used to test the homoscedasticity of the gait speed derived from APDM and GaitPy compared to the instrumented mat (GAITRite). Agreement of gait speed across multiple devices were characterized with ICC_2,1_ (two-way random effects, absolute agreement, with respect to single measurement). Pearson’s correlation coefficients were also computed to test for the consistency between gait speed estimated using different methods.

The group analysis of in-lab walk tests was performed using a linear mixed-effects regression model with repeated measures followed by ANOVA. Each participant had data from two in-lab visits. The statistical model included method (GAITRite/APDM/GaitPy), age group (younger/older) and sex (F/M) as main factors, and height and muscle mass as covariates. Random effects (participant/visit and participant/device) were also included to account for within participant variability.

The statistical testing of the at-home data was conducted using linear mixed-effects model with repeated measures followed by ANOVA. In order to account for outliers, which are upper and lower extremes in bout length, bouts that lasted <10 s or >3000 s were excluded from the analysis. Moreover, only bouts with at least four detected gait cycles were included in the analyses to ensure robust gait parameter estimation. For each participant, median gait speed was estimated per walking bout and then fed into the statistical model. Age group (younger/older), sex (F/M), and type of day (weekday/weekend) were added as main fixed factors, and height and muscle mass were added as covariates. Random effects (participant/type of day/each day) were also added to account for within participant variability. The 95^th^ percentile gait speed was summarized over all walking bouts and fed into the same linear model, excluding type of day and random effects.

In addition, the same analyses including ICC, Bland–Altman, and group analysis were repeated for each gait metric. The group analysis *p* values were corrected for multiple comparisons using false discovery rate correction.

The association between in-lab and at-home gait speed was evaluated using a linear regression model (lm in “lme4” package), using in-lab gait speed as the dependent variable, and at-home gait speed as the independent variable. The agreement between in-lab and at-home gait speed was assessed using Spearman’s rho to account for outliers.

The amount of data required for reliable estimation of gait speed under free-living conditions was determined by drawing bootstrap samples (with replacement) from the at-home data by increasing the number of consecutive steps to estimate the gait speed from 5000 to 25,000 steps, and the number of days to estimate the gait speed from one to five days. A 1000 bootstraps were performed in each subgroup, and the analysis included participants with at least total 25,000 steps (62 participants) and five days of data (65 participants), during the continuous at-home monitoring period for steps and days analyses, respectively. For each bootstrap, we computed the median gait speed and the 95^th^ percentile gait speed per participant, and then computed the ICC with respect to the gait speed estimated from the full data for that participant. Full data for a participant ranged from 6 to 15 days based on the detected gait cycles. Reliability of estimated gait speed was assessed according to the following benchmarks: ICC ≤ 0.4 indicates poor, 0.4–0.59 moderate, 0.6–0.74 good, and 0.75–1 excellent reliability^48^.

The number of at-home monitoring days required for detecting differences in gait speed of the younger and older groups was determined using bootstrapping followed by group analysis^36^. Specifically, participants with at least 100 steps per day and five days of data were included, and 1000 bootstraps were drawn for each number of days varying from one to five days. For each bootstrap, the *t*-statistic with the contrast of age group difference was compared (*t*-bootstrap, Fig. 5) with respect to the *t*-statistic of the full data (*t*-original, Fig. 5), and the proportion of *t*-bootstrap that were greater than the original *t*-statistic were used to compute the *p* value.

### Reporting summary

Further information on research design is available in the Nature Research Reporting Summary linked to this article.

## Supplementary information

Supplementary Data 1

Supplementary Data 2

Supplementary Data 3

Supplementary Data 4

Supplementary Information

Reporting Summary Checklist

Supplementary Data 5

## Data Availability

The authors declare that the data supporting the findings of this study are available in the Supplementary information files for the paper.

## Code availability

The authors declare that the pseudo code supporting the findings of this study are available in the Supplementary information files for the paper. The Gaitpy software is available at https://github.com/matt002/GaitPy.
