# Supplementary material for: Age and environment-related differences in gait in healthy adults using wearables
Source: NPJ Digit Med. 2020 Sep 30;3:127. doi: 10.1038/s41746-020-00334-y (PMC7528045; doi:10.1038/s41746-020-00334-y)
Supplement: Supplementary file 5 — Supplementary Information [file 41746_2020_334_MOESM5_ESM.pdf]

Supplementary Material:

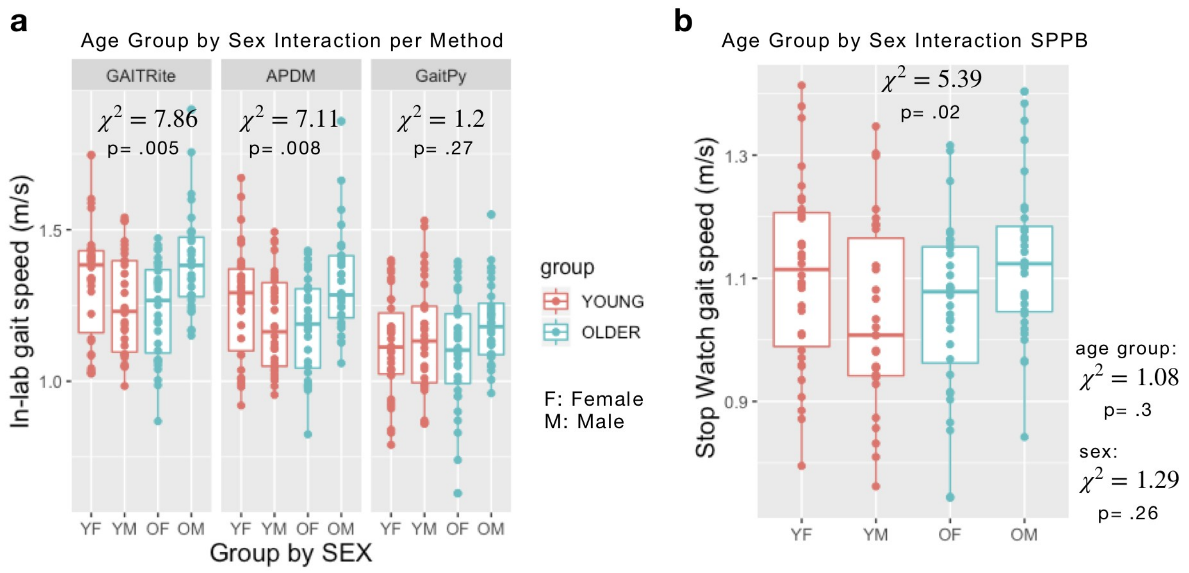

Supplementary Figure 1: Effect of sex and age group interaction in in-lab measurements. (a) GAITRite and APDM showed a significant age group by sex interaction in in-lab measurements, in which younger males were slower than younger females whereas older males walked faster than older females. (b) Similar trend was observed with uninstrumented measurements as well. The uninstrumented gait speed was computed by dividing the walking distance; i.e., 4 meters, by the average time to walk 4 meters measured by stop watch as part of standard clinic assessment; i.e., SPPB.

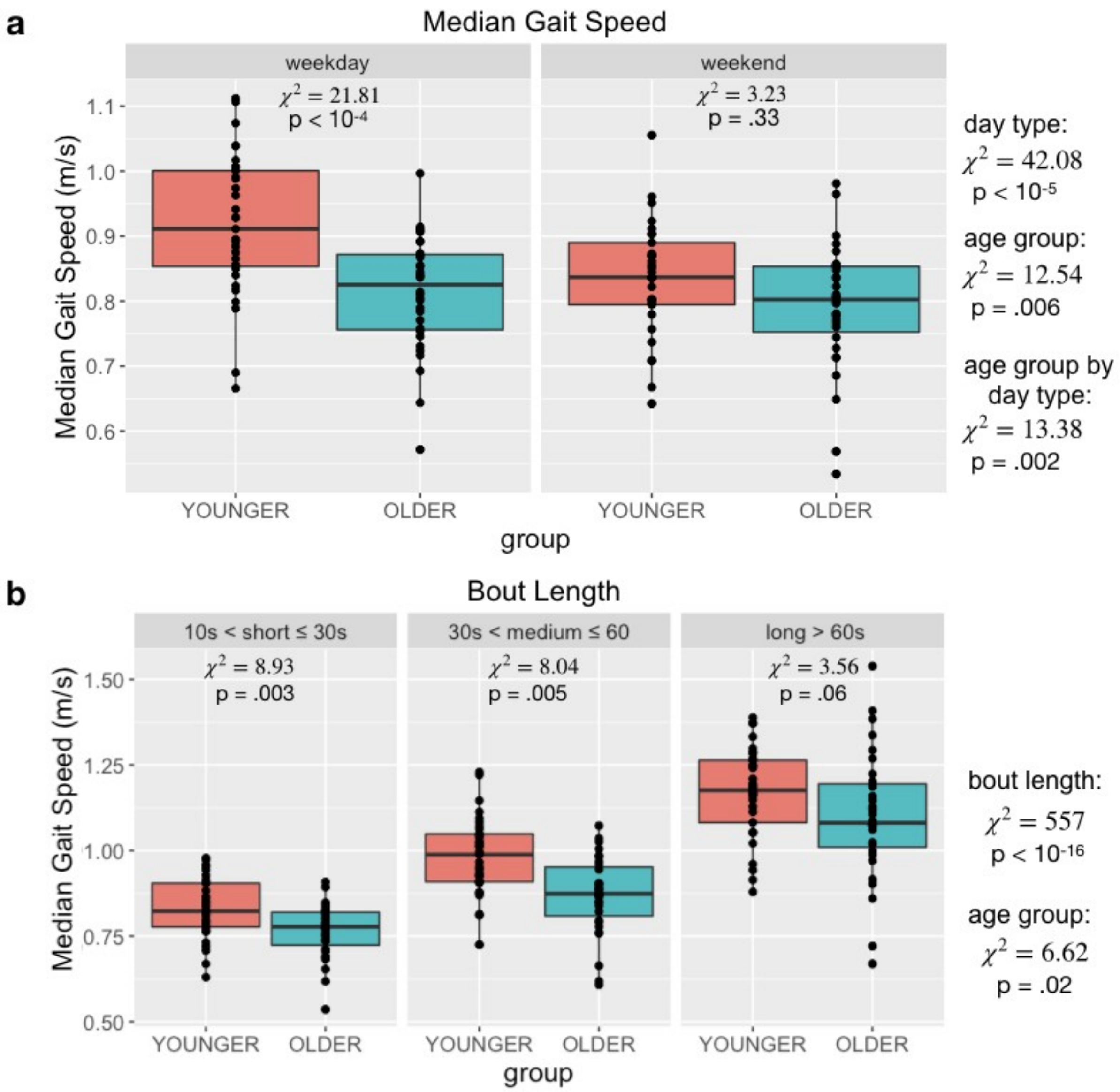

Supplementary Figure 2: Effect of day type and bout length on median gait speed. (a) Both age groups walked slower during weekends compared to weekdays, however, the age group differences were driven by weekdays, not weekends. (b) Gait speed significantly differed and increased with increasing bout lengths (effect of bout length: χ2 = 557, p < 10−16). Moreover, we observed significant or trending age group differences in all bout lengths (effect of age group: χ2 = 6.62, p =.02) with decreasing effect size; i.e., normalized χ2, with increasing bout length.

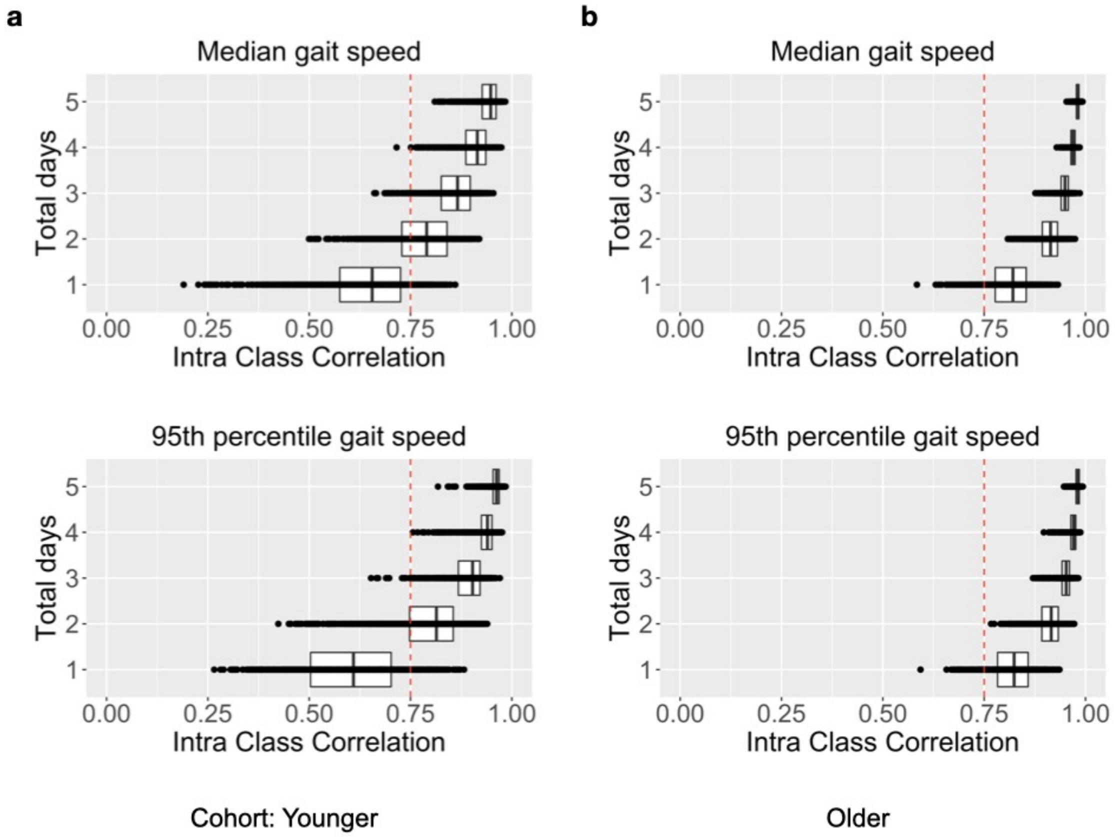

Supplementary Figure 3: Quantity of data needed to estimate at-home gait speed reliably is different between younger and older cohorts. (a) Younger participants require at least two days of data to reliably estimate at-home gait speed, whereas (b) older participants require at least 1 day of data.

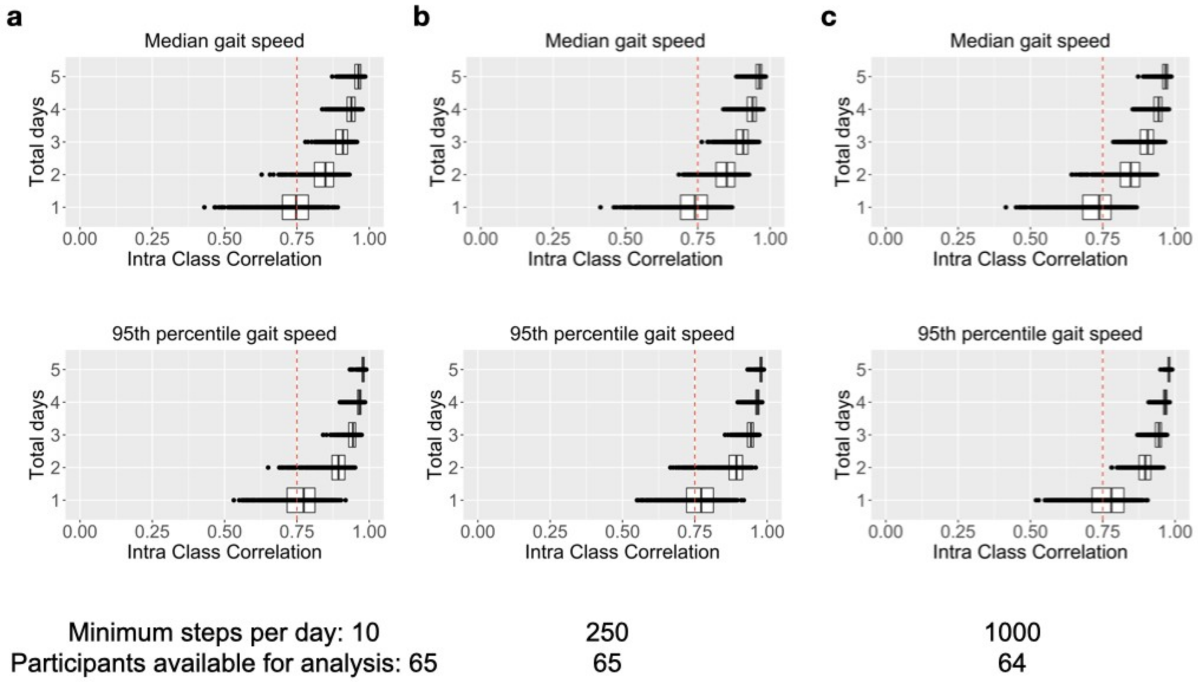

Supplementary Figure 4: Number of monitoring days required for various criteria. Varying the minimum threshold for steps per day to be considered for analysis between (a) 10, (b) 250, and (c) 1,000 does not impact the quantity of data needed to estimate at-home gait speed reliably but does reduce the participants available for analysis.

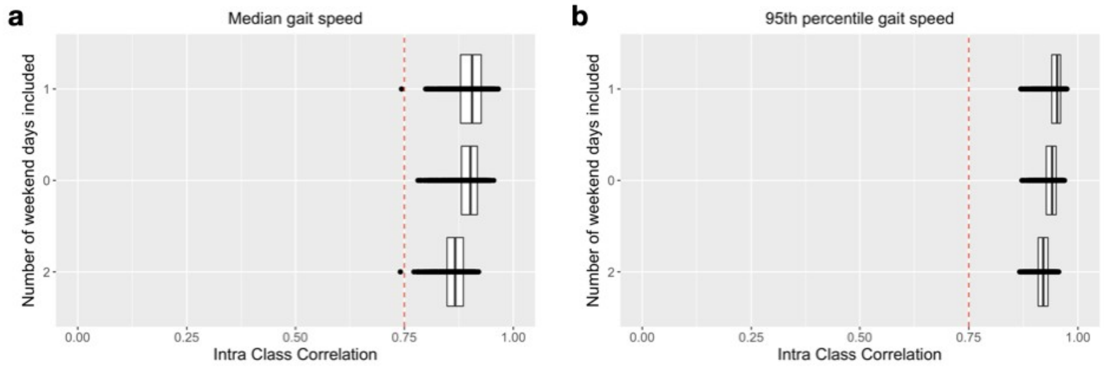

Supplementary Figure 5: Reliability of gait speed for weekends vs weekdays. Including one weekend day, but not two, out of three total days only slightly enhances reliability of estimated at-home (a) median and (b) 95th percentile gait speed. Random subsets of data that included three, two, and one weekdays out of three total days were compared to the full data set.

	GAITRite						APDM						GaitPy						Device	
	Visit 1[a]		Visit 2[a]		Age group		Visit 1[a]		Visit 2[a]		Age group		Visit 1[a]		Visit 2[a]		Age group			
	Younger	Older	Younger	Older	X^2	p	Younger	Older	Younger	Older	X^2	p	Younger	Older	Younger	Older	X^2	p	X^2	p
gait speed (m/s)	1.29 ± 0.18	1.3 ± 0.19	1.31 ± 0.19	1.33 ± 0.17	0.33	0.57	1.22 ± 0.17	1.24 ± 0.19	1.24 ± 0.18	1.27 ± 0.17	0.57	0.45	1.13 ± 0.16	1.13 ± 0.18	1.13 ± 0.19	1.15 ± 0.16	0.03	0.87	199.1	$< 10^{-16}$
swing time (s)	0.4 ± 0.03	0.39 ± 0.03	0.4 ± 0.03	0.39 ± 0.03	3.18	0.07	0.44 ± 0.03	0.43 ± 0.03	0.44 ± 0.03	0.42 ± 0.03	3.88	0.05	0.42 ± 0.03	0.41 ± 0.03	0.42 ± 0.03	0.41 ± 0.03	0.88	0.35	514.4	$< 10^{-16}$
stance time (s)	0.68 ± 0.07	0.66 ± 0.05	0.67 ± 0.07	0.65 ± 0.04	1.97	0.16	0.64 ± 0.06	0.63 ± 0.04	0.64 ± 0.06	0.62 ± 0.04	0.90	0.34	0.69 ± 0.06	0.68 ± 0.05	0.69 ± 0.05	0.67 ± 0.04	1.52	0.22	549.92	$< 10^{-16}$
double support (s)	0.27 ± 0.05	0.26 ± 0.04	0.27 ± 0.05	0.26 ± 0.04	0.47	0.49	0.2 ± 0.04	0.2 ± 0.03	0.2 ± 0.05	0.2 ± 0.03	0.17	0.69	0.28 ± 0.03	0.27 ± 0.02	0.27 ± 0.02	0.26 ± 0.02	2.16	0.14	490.45	$< 10^{-16}$
single limb support (s)	0.4 ± 0.03	0.39 ± 0.03	0.4 ± 0.03	0.39 ± 0.03	1.92	0.17	0.44 ± 0.03	0.43 ± 0.03	0.44 ± 0.03	0.42 ± 0.03	0.83	0.36	0.42 ± 0.03	0.41 ± 0.03	0.42 ± 0.03	0.41 ± 0.03	0.001	0.98	113.1	$< 10^{-16}$
step time (s)	0.54 ± 0.05	0.53 ± 0.03	0.54 ± 0.05	0.52 ± 0.03	2.31	0.13	0.54 ± 0.04	0.53 ± 0.03	0.54 ± 0.04	0.52 ± 0.03	1.78	0.18	0.56 ± 0.05	0.54 ± 0.04	0.55 ± 0.04	0.54 ± 0.04	1.39	0.24	287.82	$< 10^{-16}$
stride length (m)	1.41 ± 0.14	1.39 ± 0.19	1.42 ± 0.15	1.41 ± 0.2	0.01	0.93	1.31 ± 0.14	1.31 ± 0.19	1.32 ± 0.15	1.32 ± 0.19	0.16	0.69	1.25 ± 0.17	1.24 ± 0.18	1.24 ± 0.2	1.25 ± 0.18	0.03	0.86	183.98	$< 10^{-16}$
stride time (s)	1.08 ± 0.09	1.05 ± 0.07	1.08 ± 0.09	1.04 ± 0.07	2.56	0.11	1.08 ± 0.09	1.06 ± 0.07	1.07 ± 0.09	1.04 ± 0.07	2.04	0.15	1.11 ± 0.09	1.09 ± 0.07	1.1 ± 0.08	1.07 ± 0.07	1.43	0.23	386.87	$< 10^{-16}$

Supplementary Table 1: In-clinic gait metrics derived from instrumented mat (GAITRite), APDM 6-sensor set, and GaitPy algorithm using one lumbar mounted sensor. The common gait metrics were summarized for each visit and age group. The repeated mixed model regression showed that there is a significant effect of device on all gait metrics (Device: $p < 10^{-16}$). Posthoc analyses showed no age group differences in any of the gait metrics for any device (the p-values were not corrected for multiple comparisons for this analysis).

[a] Mean ± sd

	Age Group				Day Type				Age Group x Day Type						Sex		M.Mass		Height	
	Younger[a]	Older[a]	X^2	p	Weekday[a]	Weekend[a]	X^2	p	Younger WD[a]	Older WD[a]	Younger WE[a]	Older WE[a]	X^2	p	X^2	p	X^2	p	X^2	p
95th gait speed	1.37 ± 0.13	1.19 ± 0.17	22.59	10^{-5}											1.47	0.23	3.67	0.06	2.5	0.19
gait speed	0.9 ± 0.1	0.81 ± 0.09	12.54	0.006	0.87 ± 0.11	0.81 ± 0.1	42.08	$<10^{-5}$	0.92 ± 0.11	0.81 ± 0.09	0.83 ± 0.09	0.79 ± 0.1	13.38	0.002	0.74	1	3.102	0.4	2.76	0.24
steps	67.1 ± 20.37	55.67 ± 22.82	3.99	0.049	65.84 ± 26.46	52.3 ± 27.67	24.64	$<10^{-5}$	70.99 ± 21.8	59.97 ± 26.3	57.31 ± 26.11	47.49 ± 22.82	0.24	0.672	0.73	1	2.426	0.4	2.04	0.24
gait cycle duration	1.24 ± 0.08	1.27 ± 0.07	5.16	0.030	1.25 ± 0.08	1.28 ± 0.07	39.50	$<10^{-5}$	1.23 ± 0.08	1.27 ± 0.07	1.28 ± 0.07	1.29 ± 0.07	5.20	0.043	0.00	1	0.094	0.9	1.70	0.24
step duration	0.61 ± 0.04	0.63 ± 0.03	6.29	0.024	0.62 ± 0.04	0.64 ± 0.04	40.27	$<10^{-5}$	0.61 ± 0.04	0.63 ± 0.03	0.63 ± 0.03	0.64 ± 0.04	5.07	0.043	0.01	1	0.196	0.9	1.60	0.24
cadence	100.47 ± 6.01	97.09 ± 5.27	8.54	0.012	99.54 ± 6.17	96.65 ± 5.24	42.49	$<10^{-5}$	101.45 ± 6.36	97.55 ± 5.28	97.28 ± 4.86	96.05 ± 5.47	6.48	0.034	0.11	1	0.258	0.9	1.74	0.24
initial double support	0.15 ± 0.01	0.15 ± 0.01	10.51	0.008	0.15 ± 0.01	0.15 ± 0.01	31.47	$<10^{-5}$	0.14 ± 0.01	0.15 ± 0.01	0.15 ± 0.01	0.15 ± 0.01	4.37	0.051	0.03	1	0	1	1.83	0.24
terminal double support	0.14 ± 0.01	0.15 ± 0.01	9.42	0.010	0.15 ± 0.01	0.15 ± 0.01	24.98	$<10^{-5}$	0.14 ± 0.01	0.15 ± 0.01	0.15 ± 0.01	0.15 ± 0.01	4.70	0.047	0.02	1	0.069	0.9	2.86	0.24
double support	0.29 ± 0.01	0.3 ± 0.02	7.87	0.014	0.29 ± 0.02	0.3 ± 0.01	26.94	$<10^{-5}$	0.29 ± 0.02	0.3 ± 0.02	0.3 ± 0.01	0.3 ± 0.02	5.16	0.043	0.00	1	0.051	0.9	2.22	0.24
single limb support	0.49 ± 0.04	0.5 ± 0.03	2.44	0.119	0.49 ± 0.04	0.51 ± 0.03	32.37	$<10^{-5}$	0.48 ± 0.04	0.5 ± 0.03	0.51 ± 0.03	0.51 ± 0.03	4.14	0.053	0.00	1	0.296	0.9	0.90	0.34
stance	0.76 ± 0.04	0.78 ± 0.04	6.48	0.024	0.76 ± 0.04	0.78 ± 0.04	43.03	$<10^{-5}$	0.75 ± 0.04	0.77 ± 0.04	0.78 ± 0.04	0.79 ± 0.04	6.31	0.034	0.01	1	0.036	0.9	2.18	0.24
swing	0.47 ± 0.03	0.48 ± 0.03	5.32	0.030	0.47 ± 0.03	0.48 ± 0.03	35.80	$<10^{-5}$	0.46 ± 0.03	0.48 ± 0.03	0.48 ± 0.03	0.49 ± 0.03	3.99	0.053	0.00	1	0.29	0.9	1.37	0.26
step length	0.53 ± 0.05	0.5 ± 0.05	5.10	0.030	0.52 ± 0.06	0.5 ± 0.06	25.88	$<10^{-5}$	0.54 ± 0.06	0.5 ± 0.05	0.51 ± 0.06	0.5 ± 0.05	13.06	0.002	0.62	1	2.9380	0.4	6.84	0.06
stride length	1.07 ± 0.11	1 ± 0.1	5.32	0.030	1.04 ± 0.11	1.01 ± 0.11	25.59	$<10^{-5}$	1.08 ± 0.11	1 ± 0.09	1.03 ± 0.11	0.99 ± 0.11	12.66	0.002	0.60	1	3.001	0.4	6.89	0.06
bout length	43.88 ± 11.67	37.4 ± 11.78	4.13	0.049	42.76 ± 14.03	36.31 ± 15.29	21.10	$<10^{-5}$	45.68 ± 12.26	39.5 ± 13.33	39.41 ± 14.46	33.4 ± 12.35	0.09	0.764	0.57	1	1.816	0.5	2.11	0.24

Supplementary Table 2: The regression analysis of all gait metrics derived from the at-home monitoring data. There were age group differences for almost all gait metrics except single support time. There was also a difference between the gait observed in the weekdays compared to the weekend. There was no effect of sex or muscle mass or height (except step length). There were no interaction effects between any of the variables. All the p-values were corrected for multiple comparisons using FDR. [a] Mean ± sd WD: Weekdays, WE: Weekend, M.Mass: Muscle Mass

Supplementary Code

Below is a pseudocode R code associated with the analyses and figures and tables in the manuscript.

```
# Sample code for "Age-based effects of gait in healthy adult volunteers during in-clinic
and continuous at-home monitoring with wearable devices"
# c/o Matt D. Czech & F. Isik Karahanoglu (FikretIsik.Karahanoglu@Pfizer.com)
# August 2020, Pfizer Inc. Cambridge, MA, USA


###############################################################################
# group analyses in-clinic data model Figure 2 (device effect, group effect, device by
group effect) & Supplementary Table 1
result<-car::Anova(lmer(gait_speed~group*device*(visit+SEX) + HT + Muscle_Mass +
(1|subject/visit) + (1|subject/device) , data), type="III")

###############################################################################


###############################################################################
# group analyses at-home data model Figure 3 & Supplementary Table 2

result<-car::Anova(lmer(gait_speed~group*day_type*SEX + Muscle_Mass + HT + (1 |
Subject/day_type/Date),data), type="III")
# multiple comparisons use p.adjust, method ="fdr"

###############################################################################


###############################################################################
# Figure 5a: random sample days and compare to gait speed on all days

run_icc_day_analysis <- function(athome_data_all, min_steps, min_days, cohort,
random_samples, file_name, figure_title, percentile){
  # filter cohort
  if (!cohort == 'none'){
    athome_data_f <- athome_data_all[athome_data_all$group == cohort,]
    print(paste0('Including ',cohort,' subjects only...'))
  } else {athome_data_f <- athome_data_all}

  # calculate 95 percentile gait speed from all data
  athome_data_temp <- athome_data_f[c('subject','gait_speed', 'group')]
  dt<-data.table(athome_data_temp)
  perc_95 <- dt[, quantile(gait_speed, percentile), by = c('subject', 'group')]
  colnames(perc_95)[3] <- "gait_speed_95q"

  # remove days with less than n steps
  athome_data_f$subject_day <- paste0(athome_data_f$subject,'_', athome_data_f$days)
  steps_per_subj_day <-aggregate(athome_data_f$steps,
by=list(subject_day=athome_data_f$subject_day), FUN=sum)
  include_subj_day <- steps_per_subj_day[which(steps_per_subj_day$x >=
min_steps),c('subject_day')]

athome_data_remove_subj_day <- athome_data_f[athome_data_f$subject_day %in%
include_subj_day,]
  # remove subjects with less than n days
```

```
  day_summary <- athome_data_remove_subj_day %>% dplyr::group_by(subject, days) %>%
dplyr::summarise(length(days))
  day_counts <- ddply(day_summary, .(day_summary$subject), nrow)
  remove_days <- day_counts[which(day_counts$V1 < min_days),]
  athome_data_remove_day <-athome_data_remove_subj_day[!athome_data_remove_subj_day$subject
%in% remove_days$`day_summary$subject`,]

  # print subject removal
  print(paste0('total subjects: ', length(unique(athome_data_f$subject))))
  print(paste0('subjects with at least ', min_days, ' days with at least ', min_steps, '
steps per day: ', length(unique(athome_data_remove_day$subject))))
  if (file.exists(paste0(results_dir,file_name,".RData"))){
    load(paste0(results_dir,file_name,".RData"))
  }else{
    # subsample and run ICC
    athome_data <- athome_data_remove_day[c('subject', 'days','gait_speed', 'group')]
    ICC_results <- NULL

    for (d in 1:min_days){
      for (i in 1:random_samples){
        # random sample n days
        athome_data_temp <- athome_data %>% group_by(subject, days) %>% nest()
        athome_data_temp <- athome_data_temp %>% group_by(subject) %>% sample_n(d)
        athome_data_temp <- athome_data_temp %>% unnest(data)

        dt<-data.table(athome_data_temp[c('subject','gait_speed')])
        temp_perc_95 <- dt[, quantile(gait_speed, percentile), by = c('subject')]
        colnames(temp_perc_95)[2] <- "sample_95q"

        # generate ICC results
        merged_perc_95 <- merge(temp_perc_95,perc_95,by="subject")
        temp <-
ICC(cbind(merged_perc_95$sample_95q,merged_perc_95$gait_speed_95q),missing=TRUE)
        ICC_results <- rbind(ICC_results,c(temp$results[2,],"gait_speed_95q",d))
      }
    }

    colnames(ICC_results)[c(9,10)] <- c("metric","days")
    ICC_results_f<-tbl_df(ICC_results)
    ICC_results_f$ICC = as.double(ICC_results_f$ICC)
    ICC_results_f$`upper bound` = as.numeric(ICC_results_f$`upper bound`)
    ICC_results_f$`lower bound` = as.numeric(ICC_results_f$`lower bound`)
    ICC_results_f$days = as.numeric(ICC_results_f$days)
    save(ICC_results_f, file = paste0(results_dir,file_name,".RData"))
  }
  for (d in 1:min_days){
    ICC_d_steps <- ICC_results_f[ which(ICC_results_f$days==d),]
    print(paste0(d, ' days: MedianICC=', median(ICC_d_steps$ICC), '; MinICC=',
min(ICC_d_steps$ICC), '; MaxICC=', max(ICC_d_steps$ICC)))
  }
  print(
    ggplot(ICC_results_f, aes(x = reorder(days, ICC, FUN = median), ICC)) + geom_boxplot()
+ geom_point() + ylim(0,1) +geom_hline(yintercept=.75, linetype="dashed", color = "red") +
      ggtitle(figure_title)+ ThemeMain2 + ylab("Intra Class Correlation") + xlab("Total
days") + coord_flip()
  )
  ggsave(paste0(results_dir, file_name, ".png"))
```

```
}

# example script to run
run_icc_day_analysis(athome_data_all, 10, 5, 'none', 1000,
'concurrent_days_gait_speed_95q_10stepmin', '95th percentile gait speed', 0.95)
# run_icc_day_analysis(athome_data_all, 10, 5, 'none', 1000,
'concurrent_days_gait_speed_50q_10stepmin', 'Median gait speed', 0.5)

###############################################################################



###############################################################################
# Table 2. Bootstraping
# uses the logic http://galton.uchicago.edu/~eichler/stat24600/Handouts/bootstrap.pdf

# function that bootstrap from various days and compute t-stat for age group difference,
then rank the value of the original t-value using full data (all availbale days) --> p-
value
analyze_differences_days_p <- function(athome_data_f, min_steps, min_days, random_samples,
percentile){
  # athome_data_f['day'] <- substr(athome_data_f$bout_start_time, 1,10) # if day is not
defined yet
  athome_data_f['day'] <- substr(athome_data_f$bout_start_time, 1,10)

  # remove days with less than n steps
  athome_data_remove_subj_day <- athome_data_f %>% group_by(subject,day) %>%
filter(sum(steps)>= min_steps)

  # remove subjects with less than n days
  athome_data_remove_day <- athome_data_remove_subj_day %>% group_by(subject) %>%
filter(length(unique(day)) >= min_days)

  # print subject removal
  print(paste0('total subjects: ', length(unique(athome_data_f$subject))))
  print(paste0('subjects with at least ', min_days, ' days with at least ', min_steps, '
steps per day: ', length(unique(athome_data_remove_day$subject))))

  athome_data <- athome_data_remove_day[c('subject', 'day','gait_speed', 'group')]
  dt<-athome_data


  # subsample and run ICC
  stats_results <- NULL

  unique_days <- dt %>% group_by(subject,day,group) %>% summarise(gait_speed =
quantile(gait_speed,percentile)) %>% ungroup() %>% group_by(subject) %>% mutate(day_n =
row_number())

  mean_pergroup <- unique_days %>% group_by(subject,group) %>%
summarise(gait_speed_pergroup = mean(gait_speed)) %>% group_by(group) %>%
mutate(gait_speed_pergroup = mean(gait_speed_pergroup))
  mean_all <- unique_days %>% group_by(subject,group) %>% summarise(gait_speed_all =
mean(gait_speed)) %>% ungroup() %>% mutate(gait_speed_all = mean(gait_speed_all))

  transform_data <- left_join(unique_days,mean_pergroup,by=c("group","subject"))
  transform_data <- left_join(transform_data,mean_all,by=c("group","subject"))
```

```
  transform_data <- transform_data %>% mutate(transform = gait_speed - gait_speed_pergroup
+ gait_speed_all)
  transform_data_totest <- transform_data %>% group_by(subject,group) %>%
summarise(mean_transform=mean(transform),mean_gaitspeed = mean(gait_speed))
  t_orig <- t.test(mean_gaitspeed ~ group,transform_data_totest)$statistic
  print(paste("original t_orig is:", t_orig))

  t_stats_all <- data.frame()
  for (days in 1:min_days){
    print(days)
    t_stats <- c()
    for (i in 1:random_samples){
      # random sample n days
      athome_data_temp<-transform_data %>% group_by(subject) %>% sample_n(days,replace =
TRUE)

      athome_data_temp_totest<-athome_data_temp %>% group_by(subject,group) %>%
summarise(mean_transform=mean(gait_speed))
      t_boot <- t.test(mean_transform ~ group,athome_data_temp_totest)$statistic

      t_stats <- append(t_stats, t_boot)
    }
    t_stats_df <- data.frame(t_stats)
    t_stats_df['days'] <- days
    t_stats_df['t_proportion'] <- sum(abs(t_stats_df$t_stats) >= abs(t_orig)) /
nrow(t_stats_df)
    t_stats_all <- rbind(t_stats_all, t_stats_df)
  }
  t_results <- unique(t_stats_all[c('days','t_proportion')])
  rownames(t_results) <- NULL

  return(list(t_results,t_stats_all,t_orig))
}


# example script to run
min_steps=100 # min steps per day to be included
min_days= 5 # subjects to include with min number of days
random_samples=1000 # reduce the number of bootstraps for time
percentile=0.95 # 0.5 for median gait speed
perc_95 <- analyze_differences_days_p(athome_data_all, min_steps, min_days, random_samples,
percentile)

perc_95[[2]] %>% group_by(days) %>% summarise(max(t_stats),min(t_stats))
perc_95[[2]] %>% group_by(days) %>% summarise(mean(t_stats),sd(t_stats))

print('95 percentile gait speed')
print(perc_95[[1]])

ggplot(perc_95[[2]], aes(x = t_stats, fill = as.factor(days))) +
  xlab("t stats") +
  geom_density(alpha = .3) +
  ggtitle("95 perc gait speed - bootstraps t-stats") +
geom_vline(xintercept=perc_95[[3]],linetype="dashed",color = "red", size=1)

################################################################################
```
